# Intralobar Pulmonary Sequestration: A Case Report

**DOI:** 10.7759/cureus.46794

**Published:** 2023-10-10

**Authors:** Shawn R Flanagan, Pauravi Vasavada

**Affiliations:** 1 Diagnostic Radiology, Liberty University College of Osteopathic Medicine, Lynchburg, USA; 2 Pediatric Radiology, University Hospitals Cleveland Medical Center, Cleveland, USA

**Keywords:** congenital lesions, congenital lung malformations, broncho-pulmonary sequestration, congenital pulmonary airway malformation, intralobar pulmonary sequestration

## Abstract

Pulmonary sequestration is a congenital lung malformation characterized by a mass of nonfunctioning lung tissue that receives its arterial supply from an aberrant systemic artery. If symptomatic, most newborns present with respiratory distress. Recurrent infection is the most common presentation after the neonatal period. It is often diagnosed prenatally and is treated with elective surgical resection between ages six and twelve months. We present a case of an infant diagnosed with congenital pulmonary airway malformation prenatally revealed to be pulmonary sequestration at the age of six months, emphasizing the need for appropriate postnatal imaging.

## Introduction

Pulmonary sequestration is a rare congenital disease, representing only 0.15%-6.4% of congenital abnormalities of the lower respiratory tract [1]. It is a mass of nonfunctioning lung parenchyma with no connection to the tracheobronchial tree which receives its blood supply from one or more aberrant systemic arteries [1,2]. Pulmonary sequestration is divided into two main anatomic types: intralobar sequestration (ILS) and extralobar sequestration (ELS). ILS is the most common, representing approximately 75% of pulmonary sequestrations [1]. ILS is located within the normal lung and shares its visceral pleura, whereas ELS is outside of the normal lung and has its visceral pleura [1-3]. ELS can be located below the diaphragm, termed an infradiaphragmatic ELS [4]. Hybrid lesions occur when an ILS or ELS exists along with a congenital pulmonary airway malformation (CPAM) [5]. ILS is most often located in the left lower lobe and typically receives its blood supply from the lower thoracic aorta from one or more anomalous arteries [1]. Venous drainage is usually normal to the left atrium [1,2]. ELS is also most commonly located in the left hemithorax and receives its blood supply from the thoracic aorta [1,2]. Unlike an ILS, the venous drainage of ELS is to the systemic circulation via the azygos, vena cava, or directly to the right atrium [2,6]. Diagnosis is established by identifying a systemic vessel feeding the lung mass, which differentiates sequestration from other congenital lung malformations such as CPAM [3,6]. Here we present a case of an infant diagnosed with CPAM on prenatal ultrasound and postnatal noncontrast CT later revealed to be an ILS after contrast-enhanced imaging.

## Case presentation

**Figure 1:**
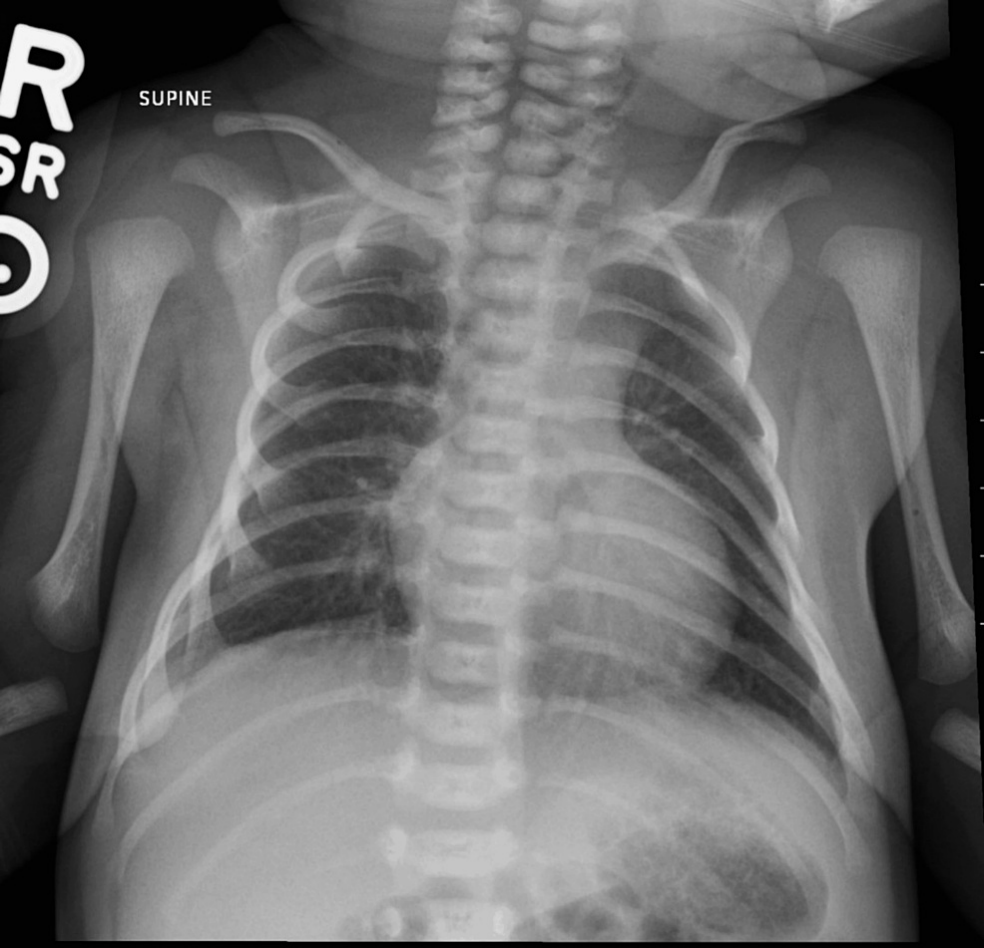
Chest radiograph does not show a discrete abnormal radiopacity that would be in keeping with a congenital pulmonary airway malformation

**Figure 2:**
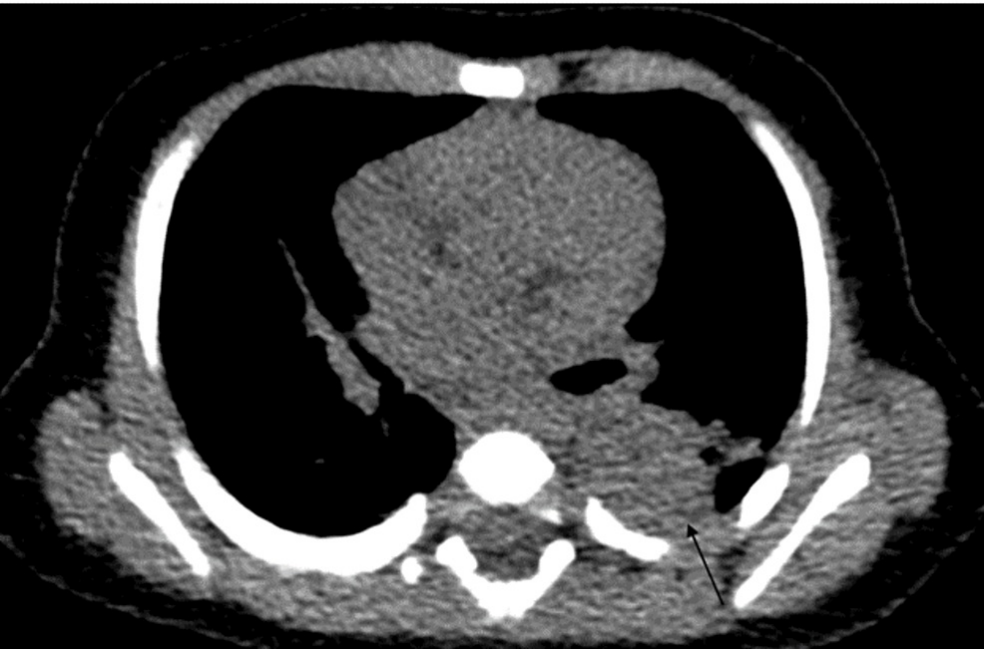
Noncontrast axial CT in a mediastinal window showing a consolidative opacity in the left lower lobe (arrow)

**Figure 3:**
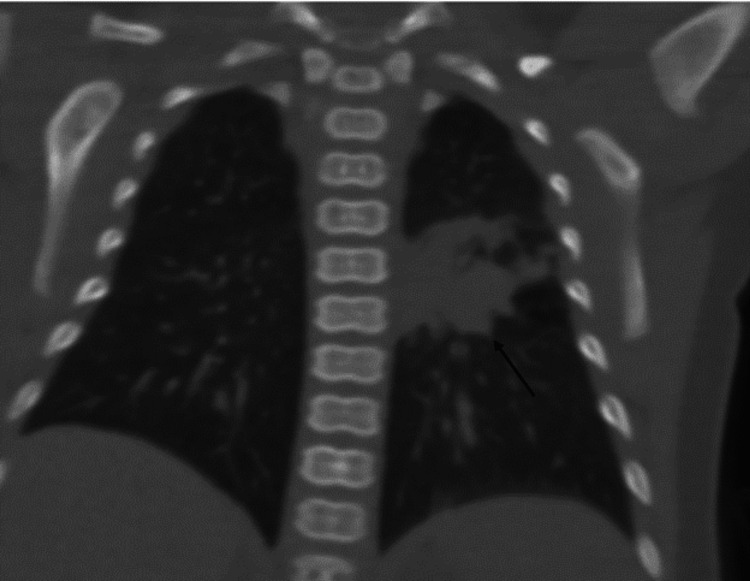
Noncontrast coronal CT showing opacity in the superior segment of the left lower lobe (arrow)

**Figure 4:**
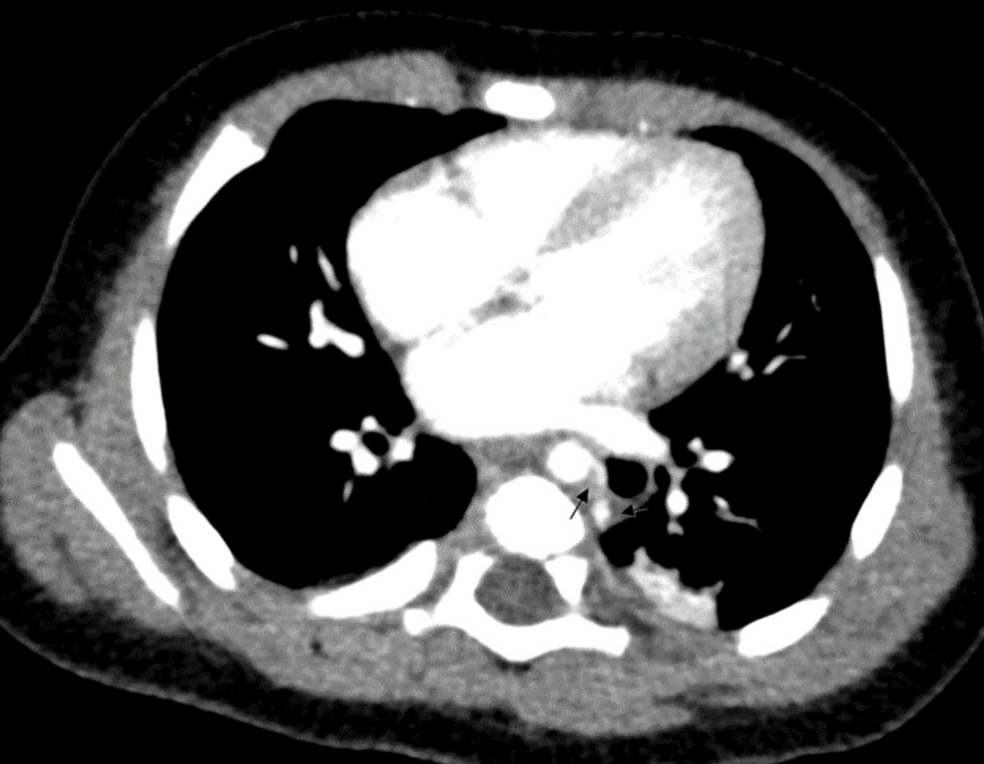
Contrast-enhanced axial CT showing the lung mass with an arterial feeder from the aorta (arrows)

**Figure 5:**
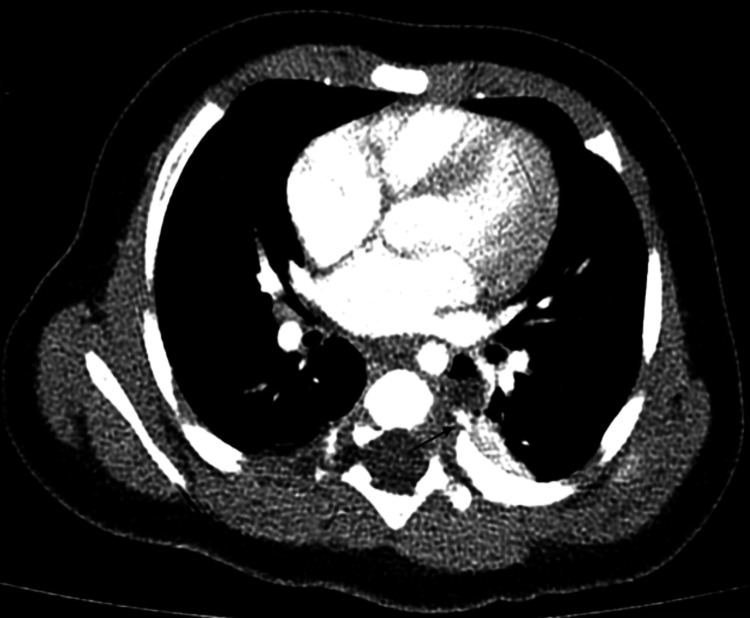
Contrast-enhanced axial CT showing an artery from the aorta feeding the lung mass (arrow)

The patient is a five-month-old female with CPAM diagnosed on prenatal ultrasound. The chest radiograph performed after delivery did not show the prenatally suggested airway malformation (Figure 1). However, the patient had a persistent cough and was treated with a course of amoxicillin/clavulanic acid and then trimethoprim-sulfamethoxazole for suspected pneumonia. Respiratory symptoms continued despite both courses of antibiotics. Noncontrast CT obtained at five months of age (Figures 2, 3) showed a consolidative opacity in the superior segment of the left lower lobe. However, no clearly defined cystic mass consistent with a CPAM was identified. Given the patient’s clinical context, the findings could be attributed to pneumonia, with underlying CPAM not excluded. Pulmonary sequestration was also considered on the differential, and follow-up CT of the chest with contrast was recommended to evaluate for vascular supply. Several weeks later a CT of the chest with contrast was obtained which revealed a vessel from the thoracic aorta feeding the mass in the left lower lobe (Figures 4, 5). Thoracoscopic left lower lobectomy was performed, which confirmed the mass was an ILS likely with an associated bronchogenic cyst. Significant oblique fissure scarring and difficult dissection of the esophagus were noted during surgery, suggesting past infection.

## Discussion

Pulmonary sequestration is a rare congenital abnormality of the lower airway consisting of a mass of nonfunctioning lung tissue receiving its blood supply from one or more aberrant systemic arteries and lacking a connection to the tracheobronchial tree [1,2]. It is a type of bronchopulmonary foregut malformation, which is a term that also includes CPAM, hybrid lesions (between CPAM and sequestration), and foregut duplication cysts [7]. The development of these congenital lung lesions is not completely known. Many etiologic theories have been proposed since Rokitansky’s fraction theory in 1861, which describes a separated portion of a normally developing lung that later becomes nonfunctional [7,8]. The current prevailing theory is that pulmonary sequestrations originate in the pseudoglandular stage (5-17 weeks gestation) of lung development [9]. This is prior to the separation of the aortic and pulmonary circulations, which could explain the spectrum of bronchopulmonary foregut malformations [9].

ILS is characterized by its location within the normal lung and lack of its own visceral pleura [1,3]. ELS is located outside the normal lung, has its own visceral pleura, and may be located below the diaphragm [1,4]. Both types of pulmonary sequestration are most commonly located within the left lower lobe, and ILS occurs primarily in the left posterior basal segment [2,10]. ILS and ELS receive their blood supply from one or more anomalous arteries from the lower thoracic or upper abdominal aorta [1,2]. The venous drainage of ILS is typically to the left atrium, although venous connections to the systemic circulation also occur [2,6]. ELS primarily drains to the systemic circulation via the azygos, hemiazygos, vena cava, or to the right atrium [6]. 

Most cases are first identified on prenatal ultrasound, although differentiating between pulmonary sequestration and CPAM is difficult due to the limited ability of ultrasound to detect a systemic arterial feeder (49% sensitivity) [11]. Up to 75% of pulmonary sequestrations decrease in size late in gestation, and many appear to resolve in utero [8,12]. Chest radiograph is the most appropriate initial imaging modality in asymptomatic neonates [13]. Advanced imaging such as CT or MRI with contrast should be obtained if the patient is symptomatic, as a definitive diagnosis requires the establishment of a lung mass with systemic arterial supply [14]. In our case, a noncontrast CT was initially obtained. This study was limited by pneumonia obscuring the underlying lung lesion and the inability to identify an aberrant systemic artery without contrast. The differential remained broad until a contrast-enhanced CT demonstrated an artery from the thoracic aorta supplying the lesion. 

Respiratory distress is the most common symptomatic presentation in the neonatal period, although most affected newborns are asymptomatic [15]. Large pulmonary sequestrations may cause respiratory distress by limiting the volume of the normal lung. Another mechanism is high-output heart failure, which may occur if a significant left-to-right shunt is established by a sequestration supplied by a large proportion of systemic blood flow [16]. After the neonatal period, ILS typically presents with recurrent infection [1,15]. Our patient with an ILS presented with persistent respiratory symptoms unresponsive to two courses of antibiotics. While ELS rarely presents with infection, more than 60% of patients with ELS have other congenital anomalies with congenital diaphragmatic hernia representing the most common co-existing anomaly [2]. Some cases after the neonatal period may present with rare complications including heart failure, massive bleeding, or torsion of the nonfunctioning lung mass. 

All patients with symptomatic pulmonary sequestration should undergo surgical excision [8,17]. Excision of an ILS, as in our patient, usually requires ligation of all vascular connections and lobectomy or segmental resection [18]. When there is a concern for high-output heart failure arterial embolization can be an effective initial therapy in lesions with a single arterial supply [19]. Optimal management for asymptomatic lesions is less clear, but the literature generally favors surgical excision [20]. Elective resection and observation are both reasonable approaches for asymptomatic lesions and low-risk (i.e., occupy less than 20% of the hemithorax and are without features of pleuropulmonary blastoma) lesions [20]. However, most evidence still favors surgery before age one in these cases. Surgery is curative, not associated with significant complications, and avoids repeated exposure to radiation in the form of monitoring radiographs and CT [17,20]. Another factor favoring excision is the lack of complete knowledge of the natural history of pulmonary sequestration complicated by a lack of commitment to continued follow-up into adulthood [20]. 

## Conclusions

Here we present a case of an intralobar pulmonary sequestration diagnosed as congenital pulmonary airway malformation on prenatal ultrasound. The diagnosis was unclear on postnatal noncontrast CT due to pneumonia obscuring the underlying lung mass. A systemic arterial feeding vessel was later identified on CT with contrast, establishing a diagnosis of pulmonary sequestration. Treatment for newborns with symptomatic pulmonary sequestration is surgical excision.
